# The State Space Subdivision Filter for Estimation on SE(2)

**DOI:** 10.3390/s21186314

**Published:** 2021-09-21

**Authors:** Florian Pfaff, Kailai Li, Uwe D. Hanebeck

**Affiliations:** Intelligent Sensor-Actuator-Systems Laboratory (ISAS), Institute for Anthropomatics and Robotics, Karlsruhe Institute of Technology (KIT), 76131 Karlsruhe, Germany; kailai.li@kit.edu (K.L.); uwe.hanebeck@kit.edu (U.D.H.)

**Keywords:** grid filter, nonlinear filtering, periodic manifold, special Euclidean group

## Abstract

The SE(2) domain can be used to describe the position and orientation of objects in planar scenarios and is inherently nonlinear due to the periodicity of the angle. We present a novel filter that involves splitting up the joint density into a (marginalized) density for the periodic part and a conditional density for the linear part. We subdivide the state space along the periodic dimension and describe each part of the state space using the parameters of a Gaussian and a grid value, which is the function value of the marginalized density for the periodic part at the center of the respective area. By using the grid values as weighting factors for the Gaussians along the linear dimensions, we can approximate functions on the SE(2) domain with correlated position and orientation. Based on this representation, we interweave a grid filter with a Kalman filter to obtain a filter that can take different numbers of parameters and is in the same complexity class as a grid filter for circular domains. We thoroughly compared the filters with other state-of-the-art filters in a simulated tracking scenario. With only little run time, our filter outperformed an unscented Kalman filter for manifolds and a progressive filter based on dual quaternions. Our filter also yielded more accurate results than a particle filter using one million particles while being faster by over an order of magnitude.

## 1. Introduction

When tracking vehicles or robots that are only capable of planar motion within rooms or on plains, their pose can be described by a two-dimensional position and a heading angle. Integrating the heading angle into the tracking is essential for realistic motion models of nonholonomic vehicles [1]. Such models may also include other angles, such as a trailer angle or the steering angle for slowly steering vehicles [2]. Estimating one’s own position and orientation is also an essential component for simultaneous localization and mapping [3].

Each position–angle pair corresponds to an element in the special Euclidean group SE(2), which is the group of two-dimensional rigid-body motions (Section 10.6.2, [4]). Quantities in SE(2) are also relevant in other settings, such as in control [5]. However, it is nontrivial to rigorously estimate states in SE(2), which is why even nowadays, some trackers rely merely on estimating the velocities along the two axes [6]. No heading angle is required to use a classical constant velocity or constant acceleration model [7]. However, such simple models generally do not adequately take the kinematics of the vehicle or robot into account.

For estimation on SE(2), it is relevant to consider that the pose involves both an angle, which is a periodic quantity on the unit circle S, and a real vector in $R^{2}$. Stacking the orientation, described by an angle in [0,2π) and the position yields a three-dimensional vector, which is an element of the Cartesian product $S\times R^{2}$. While all entries are real valued, one needs to consider the periodicity of the angle and not treat the vector as a three-dimensional Euclidean vector.

One can provide a filter for SE(2) based on the extended Kalman filter [8]. However, such filters are generally suboptimal since they cannot fully take the periodicity of the angle into account. Especially in cases with high uncertainties, the local linearization can cause a degradation of estimation accuracy. A very general filter that can be easily adapted to the SE(2) domain is the particle filter (PF) [9]. However, PFs suffer from particle degeneracy and impoverishment [10] and can be slow to converge [11]. Other general approaches that can be applied to SE(2) are the invariant extended and unscented Kalman filter [12] and the unscented Kalman filter on manifolds (UKF-M) [13]. An approach tailored explicitly to SE(2) that is based on dual quaternions was proposed in [14]. A filter based on sample reduction for scenarios in which only samples of the noise are available was presented in [15].

It is possible to make use of the property that the state can be described as a Cartesian product of a nonlinear domain and a linear domain. If we denote the part of our state describing the orientation with $x^{\omega }$ and the part describing its translation with ${\underline{x}}^{\tau }$, then we can rewrite our joint density as a product of the conditional density $f^{c}\left({\underline{x}}^{\tau }|x^{\omega }\right)$ and the marginalized density for the periodic part $f^{\omega }\left(x^{\omega }\right)$. Splitting the state up into a part that is well suited for estimation using the Kalman filter and another part for which a nonlinear filter should be applied is a technique that has become popular for PFs as Rao–Blackwellization [16] and has also found application in other filters such as the point mass filter [17]. This technique was also the foundation for the grid-based filter for SE(2) [18].

The filter we present in this paper also builds upon the idea of splitting up the state. It differs from the filter proposed in [18] in several regards. First, system models in our novel filter should be given in the form of a transition density instead of an equation of random variables. Second, no way to provide a smooth continuous density was presented in [18]. In our current paper, we consider different ways to provide a continuous density based on the parameters of our filter.

Another limitation of [18] is that the conditionals for the likelihood and the system noise all have the same parameters. In our current paper, we have no such limitation. Thus, the system and measurement noise for the position can be orientation dependent. The system noise can depend on both the current angle and the potential future angle. Especially the dependency on the current angle is important because the uncertainty in the predicted position can depend on the orientation in many applications. For the likelihood, which describes the measurement model, orientation dependency is relevant since the orientation of an object can have an influence on the error in the position measurement. Last, unlike [18], we allow for system inputs that depend on the orientation, which can be the key to solving scenarios such as the evaluation scenario in this paper.

For the nonlinear part concerning the angle, we use a grid-based filter that was first presented in [19] and later extended for arbitrary nonlinear models and generalized to arbitrary-dimensional hypertori in [20]. The filter uses trigonometric polynomials [21] to provide an interpolation of the grid values that closely matches the original continuous density even when using small numbers of grid points. Similar filters were also presented for the (hyper)sphere and hyperhemisphere [22,23]. All these filters have in common that they can be interpreted to use a subdivision of the state space into non-overlapping regions. Since the domains in the grid filters mentioned in this paragraph are bounded, they can be subdivided into areas of finite size.

For our novel filter, which we call the state space subdivision filter (S3F), we subdivide the domain $S\times R^{2}$, which can be used to describe planar poses, into a finite number of non-overlapping parts. For this, we subdivide S into the set of areas $A=\{A_{1},\ldots ,A_{n}\}$ with n∈N. Each of the *n* areas in the subdivision is in the form [a,b), with *a* and *b* in [0,2π). Note that for the unit circle, we get a valid interval even if b<a. In this case, the interval crosses the boundary of periodicity, which is located at 2π in our case. Based on the subdivision of S, the subdivision of $S\times R^{2}$ is then $\left\{A_{i}\times R^{2}|i\in \left\{1,\ldots ,n\right\}\right\}$. Please note that the focus of our explanations and derivations will be on the elements of A and that we will use the term area to refer to the subsets of the periodic domain and not subsets of the entire state space.

For the derivation of our new filter, we introduce three assumptions. For an angle α, the marginalized density $f^{\omega }\left(\alpha \right)$ and the conditional density $f^{c}\left({\underline{x}}^{\tau }|\alpha \right)$ are assumed to only depend on which area α lies in and not on the precise value of α. Furthermore, we assume that $f^{c}\left({\underline{x}}^{\tau }|\alpha \right)$ is a Gaussian density. This will allow us to use the basic ideas of the grid filter for the density describing the orientation and the Kalman filter for the conditional part. However, due to dependencies, significant adjustments are required.

**Figure 1:**
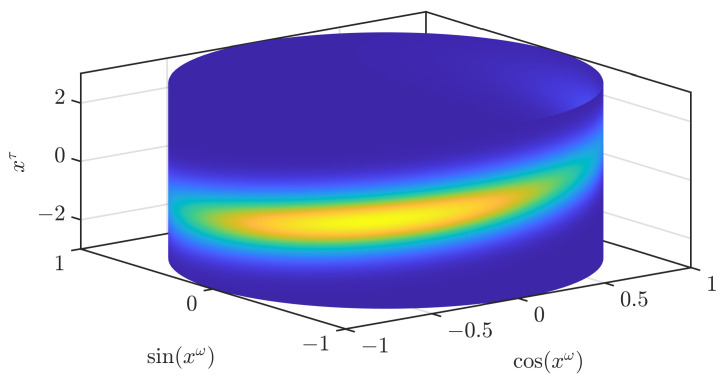
True density of the partially wrapped normal distribution.

**Figure 2:**
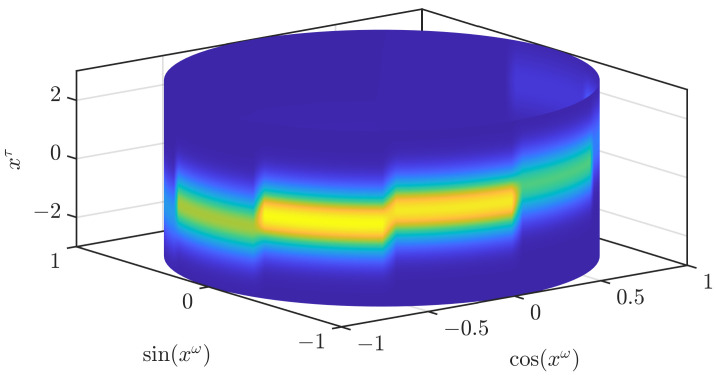
Naïve approximation of the density shown in Figure 1 based on a subdivision of the state space. 10 areas are used in this example.

While we focus on SE(2) in this paper, the S3F can be directly applied for $S\times R^{d}$ with d∈N and most key parts of our filter are not limited to $S\times R^{d}$. It can be adopted for other Cartesian products, such as of a hypertorus or hypersphere and a Euclidean domain, using grid filters for the respective domain [22,23]. To allow for easier visualization, we limit ourselves to plotting densities on S×R. This domain could, e.g., be used to estimate the rotor angle and velocity of a motor. In Figure 1, we show the density of a partially wrapped normal distribution [24] on S×R with mean ${\left[0,0\right]}^{⊤}$ and a covariance matrix with 1 in the entries on the diagonal and 0.7 in the off-diagonal entries. An illustration of all the assumptions of our filter is given in Figure 2. We will later regard how better continuous densities can be derived from the parameters of the filter.

The paper is structured as follows. In the next section, we go into detail on how we represent densities, how we approximate given densities, and how a continuous density can be provided. The update and prediction steps of the S3F are derived in Section 3. We evaluate the accuracy and run time of the S3F and other state-of-the-art filters in a simulated scenario in Section 4. A discussion, a conclusion, and an outlook are presented in Section 5.

## 2. Density Representation and Approximation

Since all areas in A are in the form of [a,b), each area is (when considering the periodicity of the domain) connected and contains more than one element. For each area $A_{i}$, we specify a grid point $\beta_{i}$ in $A_{i}$, which is in some way representative of the area. For this, we define the topology-aware distance (Section 2.3.4, [25])

(1) $$d_{0}\left(\alpha_{1},\alpha_{2}\right)=\min\left(\left|\alpha_{1}-\alpha_{2}\right|,2\pi -\left|\alpha_{1}-\alpha_{2}\right|\right)$$

for two angles $\alpha_{1}$ and $\alpha_{2}$.

We place the grid point $\beta_{i}$ such that it is in $A_{i}$ and has the same distance to *a* and *b*. To simplify the filter, we only consider subdivisions of the periodic domain into areas of equal size. Hence, for *n* regions, each area is of size $\frac{2\pi }{n}$. With all these specifications, the subdivision only depends on the starting point of the first region when A is always sorted such that the next region after [a,b) is [b,c). Without loss of generality, we say the first region is the region in which 0 lies. Thus, the starting point of the first region must lie in $\left(2\pi -\frac{2\pi }{n},0\right]$.

In our implementation of the filter, we choose the subdivision that leads to the first grid point to be at 0. In this subdivision, the first area starts at $2\pi -\frac{\pi }{n}$. Thus, assuming n>3, we obtain the subdivision $A=\{\left[2\pi -\frac{\pi }{n},\frac{\pi }{n}\right),\left[\frac{\pi }{n},3\;\cdot \;\frac{\pi }{n}\right),\left[3\;\cdot \;\frac{\pi }{n},5\;\cdot \;\frac{\pi }{n}\right),\ldots ,\left[2\pi -3\;\cdot \;\frac{\pi }{n},2\pi -\frac{\pi }{n}\right)\}$ and the grid points $B=\{0,\frac{2\pi }{n},\frac{4\pi }{n},\ldots ,\left(n-2\right)\;\cdot \;\frac{\pi }{n}\}$. We use this subdivision in our filter because all grid points can be represented as $2\pi \frac{k}{n}$ with k∈{0,…,n−1}.

We proceed by providing the full details on the assumptions of our filters that we briefly summarized in the introduction. If all assumptions hold, all densities and likelihoods involved can be represented accurately and the update step will be precise (as we later see, the prediction step involves additional assumptions and approximations). To each grid point $\beta_{i}$, we assign a grid value, which we denote by $\gamma_{i}$. There are different ways to interpret the grid values [23]. However, we will not go into detail on this in our current paper and merely focus on the interpretation that $\gamma_{i}$ describes the function value of $f^{\omega }\left(\;\cdot \;\right)$ in the area $A_{i}$.

This leads us to the assumptions (A1)–(A3) that underlie the density representation in this paper.

(A1) The function value in each area is identical to the corresponding grid value, i.e., $\forall \alpha \in A_{i}:f^{\omega }\left(\alpha \right)=\gamma_{i}$.
(A2) The conditional density $f^{c}\left({\underline{x}}^{\tau }|\alpha \right)$ is the same for all $\alpha \in A_{i}$ (we denote it by $f_{i}^{c}\left({\underline{x}}^{\tau }\right)$).
(A3) The conditional density $f^{c}\left({\underline{x}}^{\tau }|\alpha \right)$ is Gaussian for every considered α.

By combining assumptions (A2) and (A3), we get the assumption that every $f_{i}^{c}\left({\underline{x}}^{\tau }\right)$ is Gaussian. We shall call the mean of that Gaussian ${\underline{\mu }}_{i}$ and the covariance $C_{i}$, and write the pdf of the normal distribution $N({\underline{\mu }}_{i},C_{i})$ at ${\underline{x}}^{\tau }$ as $f_{N}\left({\underline{x}}^{\tau };{\underline{\mu }}_{i},C_{i}\right)$. The combination of all three assumptions leads to a joint density consisting of scaled Gaussians that are extruded along the periodic dimension, as can be seen in Figure 2. For densities encountered in many scenarios, the assumptions (A1)–(A3) do not hold. By approximating densities and likelihoods based on these assumptions, we introduce errors. However, the errors tend to get smaller with an increasing number of regions *n*.

Based on these assumptions, we can now derive a formula for the parametric density. We start with the first two assumptions and then also introduce the third one, leading to

(2) $$\begin{array}{cc} f\left({\underline{x}}^{\tau },x^{\omega };{\left\{(\gamma_{i},{\underline{\mu }}_{i},C_{i})\right\}}_{i=1}^{n}\right)\overset{\left(\mathrm{A1})\&(\mathrm{A2}\right)}{=} & \sum_{i=1}^{n}𝟙\left\{x^{\omega }\in A_{i}\right\}\gamma_{i}f_{i}^{c}\left({\underline{x}}^{\tau }\right) \end{array}$$

(3) $$\begin{array}{cc} \overset{\left(A3\right)}{=}\;\;\;\; & \sum_{i=1}^{n}𝟙\left\{x^{\omega }\in A_{i}\right\}\gamma_{i}f_{N}\left({\underline{x}}^{\tau };{\underline{\mu }}_{i},C_{i}\right)\;, \end{array}$$

with ${\left\{\left(\gamma_{i},{\underline{\mu }}_{i},C_{i}\right)\right\}}_{i=1}^{n}$ denoting the set containing all tuples $\left(\gamma_{i},{\underline{\mu }}_{i},C_{i}\right)$ for i∈{1,…,n} and 𝟙 being an indicator function that is 1 when the expression in braces is true and 0 otherwise. Throughout this paper, we will use that the marginalized density for the periodic part does not depend on the parameters of the Gaussians. This can be proven via

(4) $$\begin{array}{cc} f^{\omega }\left(x^{\omega };{\left\{(\gamma_{i},{\underline{\mu }}_{i},C_{i})\right\}}_{i=1}^{n}\right) & =\int_{R^{2}}\sum_{i=1}^{n}𝟙\left\{x^{\omega }\in A_{i}\right\}\gamma_{i}f_{N}\left({\underline{x}}^{\tau };{\underline{\mu }}_{i},C_{i}\right)d{\underline{x}}^{\tau }\\ \; & =\sum_{i=1}^{n}𝟙\left\{x^{\omega }\in A_{i}\right\}\gamma_{i}\int_{R^{2}}f_{N}\left({\underline{x}}^{\tau };{\underline{\mu }}_{i},C_{i}\right)d{\underline{x}}^{\tau }\\ \; & =\sum_{i=1}^{n}𝟙\left\{x^{\omega }\in A_{i}\right\}\gamma_{i}\;. \end{array}$$

Thus, we can also write the marginal as $f^{\omega }\left(x^{\omega };\underline{\gamma }\right)$, with $\underline{\gamma }$ being the vector containing all grid values.

In the remainder of this section, we first address how we can obtain the parameters for a given density. The second subsection addresses how we can calculate the parameter of a normalized density from the parameters describing an unnormalized one. In the third subsection, we describe other ways to provide continuous densities based on the same parameter set. This only serves as a means to obtain smoother densities than the one in Figure 2 and is not used in the prediction and update steps.

### 2.1. Deriving Parameters for a Given Density

We first discuss the case when the position and orientation are independent in the next paragraph and then proceed with the general case. The position and orientation may be independent for the initial prior density or certain likelihood functions. Note that the orientation and position usually do not stay independent throughout the prediction and filter steps. If they did, separate filters could be used and the S3F would not be required.

If the position and orientation are independent, we have $f^{c}\left({\underline{x}}^{\tau }|x^{\omega }\right)f^{\omega }\left(x^{\omega }\right)=$$f^{\tau }\left({\underline{x}}^{\tau }\right)f^{\omega }\left(x^{\omega }\right)$. For the periodic part $f^{\omega }\left(x^{\omega }\right)$, the function values at the grid points B are stored in the vector of grid values $\underline{\overset{˘}{\gamma }}$, as is done by the grid filter for the circle [19]. If $f^{\tau }\left({\underline{x}}^{\tau }\right)$ is a Gaussian density, all ${\underline{\mu }}_{i}$ and $C_{i}$ are set to its parameters (the parameters are the same for each area due to the independence of $f^{\tau }\left({\underline{x}}^{\tau }\right)$ and $f^{\omega }\left(x^{\omega }\right)$). If $f^{\tau }\left({\underline{x}}^{\tau }\right)$ is not a Gaussian, we can approximate it with a Gaussian, e.g., via moment matching.

We now consider the case in which the two parts are dependent. We do not require the full density $f^{\omega }\left(x^{\omega }\right)$ but only the value of the density at the grid points. The values can be determined via

(5) $${\overset{˘}{\gamma }}_{i}=f^{\omega }\left(\beta_{i}\right)=\int_{R^{2}}f\left({\underline{x}}^{\tau },\beta_{i}\right)d{\underline{x}}^{\tau }\;,$$

which involves a 2-D integral for each of the *n* areas. Based on the grid values, we obtain the conditional density

(6) $$f_{i}^{c}\left({\underline{x}}^{\tau }\right)=f^{c}\left({\underline{x}}^{\tau }|\beta_{i}\right)=\frac{f({\underline{x}}^{\tau },\beta_{i})}{f^{\omega }\left(\beta_{i}\right)}=\frac{1}{{\overset{˘}{\gamma }}_{i}}f\left({\underline{x}}^{\tau },\beta_{i}\right)$$

for every region. If the parameters of the Gaussian are not directly available, we can use

(7) $${\underline{\mu }}_{i}=\int_{R^{2}}{\underline{x}}^{\tau }f_{i}^{c}\left({\underline{x}}^{\tau }\right)d{\underline{x}}^{\tau }$$

to obtain the mean vector of the Gaussian for the area *i* and

(8) $$c_{i,j,k}=\int_{R^{2}}\left(x_{i}^{\tau }-\mu_{i,j}\right)\left(x_{j}^{\tau }-\mu_{j,k}\right)f_{i}^{c}\left({\underline{x}}^{\tau }\right)d{\underline{x}}^{\tau }$$

for the entry in column *j* and row *k* of the covariance matrix for area *i*. In total, we need *n* 2-D integrals for the grid values, 2n 2-D integrals for all components of the means for all areas, and 3n 2-D integrals for all components of the covariance matrices. Due to the expensive integrals, determining the parameters should be avoided during prediction and update steps. However, the integral formulae can be useful for, e.g., transforming a prior density offline.

The grid values are guaranteed to be nonnegative since they are function values of $f^{\omega }\left(x^{\omega }\right)$ or integrals of function values of $f({\underline{x}}^{\tau },x^{\omega })$. However, it cannot be guaranteed that any of the grid values is positive. If none is positive, using a higher resolution or a different filter should be considered. Furthermore, the density may not be normalized. If $f^{\omega }\left(x^{\omega };\underline{\overset{˘}{\gamma }}\right)$ is normalized, then the joint density based on the parameters is also normalized since the Gaussian for the translation is inherently normalized. However, it may happen that $f^{\omega }\left(x^{\omega };\underline{\overset{˘}{\gamma }}\right)$ is unnormalized. This is the reason why we wrote the vector as $\underline{\overset{˘}{\gamma }}$, and we will continue using this decorator to denote that the density described by the vector may be unnormalized. The final step of our approximation procedure is to determine the parameters of the normalized density. This is done as described in the next subsection.

### 2.2. Normalization

For normalizing a density, it is crucial how a parametric density as described by (3) can be scaled. It is trivial to see that if one scales every grid value with a factor λ, all function values are scaled by λ. The factor by which we need to scale the function is the reciprocal of the integral since scaling it with this value will lead to a function that integrates to one. We first derive the formula for the integral of the parametric density. For this, we use (4) to obtain

(9) $$\frac{1}{\lambda }=\int_{R^{2}}\int_{S}f\left({\underline{x}}^{\tau },x^{\omega };{\left\{({\overset{˘}{\gamma }}_{i},{\underline{\mu }}_{i},C_{i})\right\}}_{i=1}^{n}\right)dx^{\omega }d{\underline{x}}^{\tau }=\int_{S}f^{\omega }\left(x^{\omega };\underline{\overset{˘}{\gamma }}\right)dx^{\omega }\;.$$

With this formula, we can confirm that the joint density is normalized when $f^{\omega }\left(x^{\omega };\underline{\overset{˘}{\gamma }}\right)$ is normalized.

We can now proceed using the assumption (A1) to get

(10) $$\frac{1}{\lambda }=\int_{S}\sum_{i=1}^{n}𝟙\left\{x^{\omega }\in A_{i}\right\}{\overset{˘}{\gamma }}_{i}dx^{\omega }=\sum_{i=1}^{n}{\overset{˘}{\gamma }}_{i}\left|A_{i}\right|=\frac{2\pi }{n}\sum_{i=1}^{n}{\overset{˘}{\gamma }}_{i}=\frac{2\pi }{n}{∥\underline{\overset{˘}{\gamma }}∥}_{1}$$

in which $\left|A_{i}\right|$ is the size of the respective subset of the periodic domain $A_{i}$ and ${∥\;\cdot \;∥}_{1}$ denotes the 1-norm of the vector. Hence, the vector describing a normalized density can be obtained via

(11) $$\underline{\gamma }=\underline{\overset{˘}{\gamma }}/\left(\frac{2\pi }{n}{∥\underline{\overset{˘}{\gamma }}∥}_{1}\right)\;.$$

### 2.3. Providing Continuous Densities

One way to provide a continuous density is to use (3), which integrates all assumptions on which we base the prediction and update steps. Since modulo arithmetics can be employed to find the relevant area in O(1) and evaluating the corresponding Gaussian is also in O(1), evaluating the density is in O(1). We can also apply (3) for Cartesian products of $R^{d}$ with other periodic (or bounded) domains.

**Figure 3:**
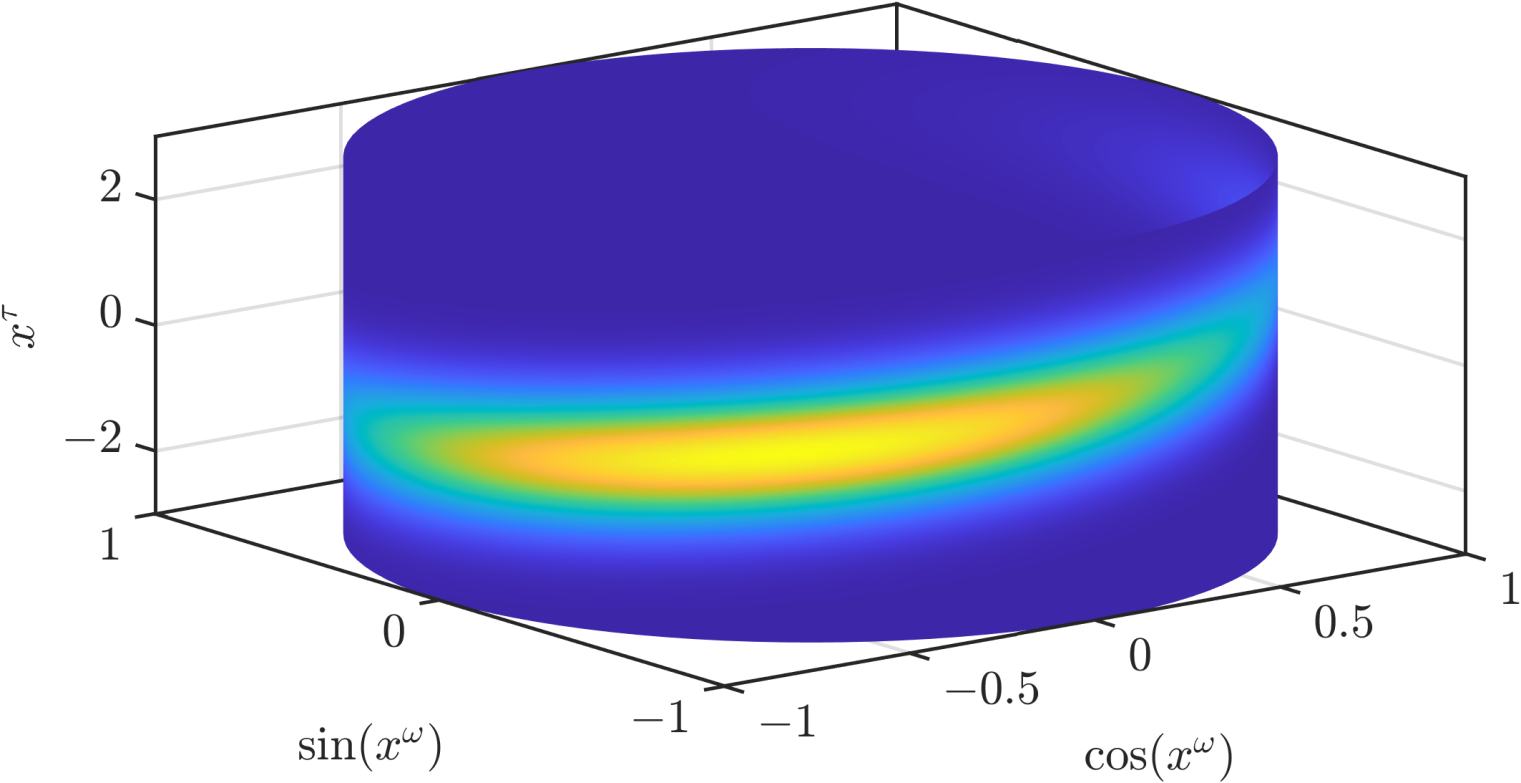
Continuous density based on a trigonometric polynomial for the periodic part.

In Figure 2, we illustrate how an approximation according to (3) looks like. While the result clearly resembles the original density, it is not an accurate approximation. In our first modification to obtain a density that is closer to the original density, we drop the assumption (A1). In the paper on the grid filter [19] that our current paper is based on, two ways to interpolate a periodic function based on function values on a grid are given. The first one does not ensure the nonnegativity of the density, while the other one does. We use the interpolation that ensures the nonnegativity and apply it to the periodic part $f^{\omega }\left(x^{\omega }\right)$. As stated previously, the joint density is normalized if the density for the periodic part is normalized. Thus, the normalization of the density when changing the interpolation scheme for the periodic part can be directly concluded from the normalization of the density for the periodic part, which is proven in the appendix of [19]. In Figure 3, we show the result when the conditional densities are unchanged. The density is now much smoother but still shows significant changes at the borders of the different areas and is not yet an accurate approximation of the original density.

To obtain smooth transitions between the areas, we drop the assumption (A2) and consider potentially (different) conditionals within each area. Since we only have parameters of *n* Gaussians, we explore ways to generate other conditionals based on these parameters. For the conditional at a grid point $\beta_{i}$, the parameters ${\underline{\mu }}_{i}$ and $C_{i}$ should always be used. For any other point α, we determine the points $\beta_{i}$ and $\beta_{i+1}$ (for i=n, i+1 shall be 1) that are closest to α in respect to the distance $d_{0}$. Then, we calculate a value that describes how much of the distance between two consecutive grid points (which is 2π/n) lies between $\beta_{i}$ and α using

(12) $$\eta \left(\alpha \right)=\frac{d(\alpha ,\beta_{i})}{2\pi n}\;.$$

The corresponding factor for $\beta_{i+1}$ is 1−η(α).

**Figure 4:**
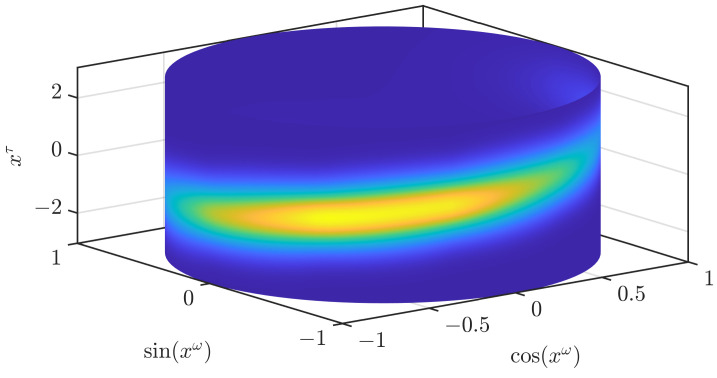
Continuous density obtained by the use of a trigonometric polynomial for the periodic part and distance-based mixtures of Gaussians for the linear part.

As the first of two alternative ways to obtain other conditionals, we propose to also drop (A3) and use the Gaussian mixture

(13) $$f^{c}\left({\underline{x}}^{\tau }|\alpha \right)=\eta f_{i}^{c}\left({\underline{x}}^{\tau }\right)+\left(1-\eta \right)f_{i+1}^{c}\left({\underline{x}}^{\tau }\right)$$

instead of the Gaussian at the closest grid point. This leads to a much smoother result, as can be seen in Figure 4. However, $f^{c}\left({\underline{x}}^{\tau }|\alpha \right)$ may be multimodal for α∉B.

Our second option is to preserve (A3) and use a convex combination of the Gaussians’ parameters as the parameters of a new Gaussian according to

(14) $$\begin{array}{cccc} \underline{\mu }\left(\alpha \right) & =\eta \left(\alpha \right){\underline{\mu }}_{i}+\left(1-\eta \left(\alpha \right)\right){\underline{\mu }}_{i+1}\;, & C(\alpha ) & =\eta \left(\alpha \right)C_{i}+\left(1-\eta \left(\alpha \right)\right)C_{i+1}\;. \end{array}$$

This guarantees that the conditional density of $N(\underline{\mu }\left(\alpha \right),C\left(\alpha \right))$ is always unimodal. In the formula for the covariance, we use that the convex combination of symmetric positive definite matrices is always symmetric positive definite. While this approach leads to a good approximation quality, we did not observe a clear superiority over using (13). For our considered example, the plot is visually indistinguishable from Figure 4, which is why we have omitted it. This approach may be more expensive in practical implementations since a different covariance matrix is used for every point at which the pdf is to be evaluated. The other two approaches involve evaluating at most *n* differently parameterized Gaussian densities, regardless of the number of points at which the pdf should be evaluated.

## 3. Filter Derivation

In this section, we derive the update and prediction steps of the S3F. A formula that we will require in this section is the fundamental Gaussian identity (App. D, [26])

(15) $$\begin{array}{r} f_{N}\left(M\underline{x};{\underline{\mu }}^{r},C^{r}\right)f_{N}\left(\underline{x};{\underline{\mu }}^{s},C^{s}\right)=f_{N}\left({\underline{\mu }}^{r};M{\underline{\mu }}^{s},C^{r}+MC^{s}M^{⊤}\right)f_{N}\left(\underline{x};{\underline{\mu }}^{q},C^{q}\right),\\ C^{q}={\left(M^{⊤}{\left(C^{r}\right)}^{-1}M+{\left(C^{s}\right)}^{-1}\right)}^{-1},\;{\underline{\mu }}^{q}=C^{q}\left(M^{⊤}{\left(C^{r}\right)}^{-1}{\underline{\mu }}^{r}+{\left(C^{s}\right)}^{-1}{\underline{\mu }}^{s}\right)\;, \end{array}$$

which can be applied for symmetric positive definite matrices $C^{r}$ and $C^{s}$, the matrix M, and vectors ${\underline{\mu }}^{r}$, ${\underline{\mu }}^{s}$, and $\underline{x}$ with compatible sizes.

### 3.1. Update Step

The update step is performed to improve our parameters based on measurements. For simplicity, we say a single measurement is obtained at time step *t* and denote it by ${\underline{\hat{z}}}_{t}$. In our filter, we work with measurement models in the form of a likelihood function $L_{{\underline{\hat{z}}}_{t}}\left({\underline{x}}_{t}^{\tau },x_{t}^{\omega }\right)$. The likelihood function (which is not necessarily a density) describes the probability that the measurement ${\underline{\hat{z}}}_{t}$ is obtained when the state is $\left[{\underline{x}}_{t}^{\tau },x_{t}^{\omega }\right]$ (we use ; in brackets to denote that a vector is stacked vertically). The update step is based on Bayes’ rule, leading to

(16) $$\begin{array}{cc} f_{t}^{e}\left({\underline{x}}_{t}^{\tau },x_{t}^{\omega }|{\underline{\hat{z}}}_{1},\ldots ,{\underline{\hat{z}}}_{t}\right) & =\frac{L_{{\underline{\hat{z}}}_{t}}\left({\underline{x}}_{t}^{\tau },x_{t}^{\omega }\right)f_{t}^{p}\left({\underline{x}}_{t}^{\tau },x_{t}^{\omega }|{\underline{\hat{z}}}_{1},\ldots ,{\underline{\hat{z}}}_{t-1}\right)}{\int_{S}\int_{R^{2}}L_{{\underline{\hat{z}}}_{t}}\left({\underline{x}}_{t}^{\tau },x_{t}^{\omega }\right)f_{t}^{p}\left({\underline{x}}_{t}^{\tau },x_{t}^{\omega }|{\underline{\hat{z}}}_{1},\ldots ,{\underline{\hat{z}}}_{t-1}\right)\;d{\underline{x}}_{t}^{\tau }dx_{t}^{\omega }}\\ \; & \propto \underset{{\overset{˘}{f}}_{t}^{e}\left({\underline{x}}_{t}^{\tau },x_{t}^{\omega }|{\underline{\hat{z}}}_{1},\ldots ,{\underline{\hat{z}}}_{t}\right)}{\underbrace{L_{{\underline{\hat{z}}}_{t}}\left({\underline{x}}_{t}^{\tau },x_{t}^{\omega }\right)f_{t}^{p}\left({\underline{x}}_{t}^{\tau },x_{t}^{\omega }|{\underline{\hat{z}}}_{1},\ldots ,{\underline{\hat{z}}}_{t-1}\right)}}\;. \end{array}$$

with $f_{t}^{e}$ denoting the posterior and $f_{t}^{p}$ the prior density. We now focus on obtaining the unnormalized prior ${\overset{˘}{f}}_{t}^{e}$, which we can easily normalize, as explained in Section 2.2.

To simplify the notation, we omit all time indices (in the update step, it is always *t*) except in the index of the state and measurement. We also omit conditioning on measurements for additional brevity (refer to (16) to see which density is conditioned on which measurements). In the previous prediction step (or the initialization), we obtained the prior density

(17) $$\begin{array}{cc} f^{p}\left({\underline{x}}_{t}^{\tau },x_{t}^{\omega },{\left\{(\gamma_{j},{\underline{\mu }}_{j}^{p},C_{j}^{p})\right\}}_{j=1}^{n}\right) & =\sum_{j=1}^{n}𝟙\left\{x_{t}^{\omega }\in A_{j}\right\}\gamma_{j}^{p}f_{N}\left({\underline{x}}_{t}^{\tau };{\underline{\mu }}_{j}^{p},C_{j}^{p}\right)\;. \end{array}$$

For the likelihood function, we also introduce the assumptions (A1) that $L_{{\underline{\hat{z}}}_{t}}^{\omega }\left(\alpha \right)=\gamma_{i}$ for all $\alpha \in A_{i}$, (A2) that $L_{{\underline{\hat{z}}}_{t}}^{c}\left({\underline{x}}_{t}^{\tau }|x_{t}^{\omega }=\alpha \right)=L_{{\underline{\hat{z}}}_{t}}^{c}\left({\underline{x}}_{t}^{\tau }|x_{t}^{\omega }=\beta_{i}\right)$ for all $\alpha \in A_{i}$, and (A3) that all conditionals are Gaussians, leading to

(18) $$\begin{array}{cc} L_{{\underline{\hat{z}}}_{t}}\left({\underline{x}}_{t}^{\tau },x_{t}^{\omega };{\left\{(\gamma_{i};{\underline{\mu }}_{i}^{L},C_{i}^{L})\right\}}_{i=1}^{n}\right) & \overset{\left(\mathrm{A1})\&(\mathrm{A2}\right)}{=}\sum_{i=1}^{n}𝟙\left\{x_{t}^{\omega }\in A_{i}\right\}\gamma_{i}L_{{\underline{\hat{z}}}_{t}}^{c}\left({\underline{x}}_{t}^{\tau }|x_{t}^{\omega }=\beta_{i}\right)\\ & \;\;\;\overset{\left(A3\right)}{=}\;\;\;\;\sum_{i=1}^{n}𝟙\left\{x_{t}^{\omega }\in A_{i}\right\}\gamma_{i}f_{N}\left({\underline{x}}_{t}^{\tau };{\underline{\mu }}_{i}^{L},C_{i}^{L}\right)\;. \end{array}$$

Since the assumptions may not be fulfilled in general, $L_{{\underline{\hat{z}}}_{t}}\left({\underline{x}}_{t}^{\tau },x_{t}^{\omega };{\left\{(\gamma_{i};{\underline{\mu }}_{i}^{L},C_{i}^{L})\right\}}_{i=1}^{n}\right)$ will generally not be equal to the original likelihood $L_{{\underline{\hat{z}}}_{t}}\left({\underline{x}}_{t}^{\tau },x_{t}^{\omega }\right)$. We assume we have the grid values $\gamma_{i}=L_{{\underline{\hat{z}}}_{t}}^{\omega }\left(\beta_{i}\right)$ or have the marginalized density for the periodic part $L_{{\underline{\hat{z}}}_{t}}^{\omega }\left(x_{t}^{\omega }\right)$ and can evaluate it at any point. If integrals are required to determine (or approximate) any of the parameters, this has a significant negative impact on the run times. However, in many settings (see, e.g., our evaluation scenario in Section 4.1) integrals are not required to obtain the likelihood function.

The likelihood function is special in multiple ways. First, while it is nonnegative everywhere, it may not be and does not need to be normalized. The multiplication of the likelihood and the prior density is generally unnormalized, and thus, a normalization step is anyway required at the end of the update step. Second, the likelihood may only depend on a certain part of the state. If the likelihood does not depend on the linear part, one can just update the grid values as in the regular grid filter [19] without adapting the parameters of the Gaussians. If the likelihood does not depend on the orientation part, we set all $\gamma_{i}$ to 1. It is important to still perform the update step as described in this subsection and not merely perform Kalman filter updates for updating the Gaussians’ parameters in each region. This will become evident in our derivation.

We first introduce the assumptions (A1) and (A2) to obtain

(19) $$\begin{array}{cc} {\overset{˘}{f}}^{e}\left({\underline{x}}_{t}^{\tau },x_{t}^{\omega }\right)\overset{\left(\mathrm{A1})\&(\mathrm{A2}\right)}{=} & \left(\sum_{i=1}^{n}𝟙\left\{x_{t}^{\omega }\in A_{i}\right\}\gamma_{i}^{L}L_{{\underline{\hat{z}}}_{t}}^{c}\left({\underline{x}}_{t}^{\tau }|x_{t}^{\omega }=\beta_{i}\right)\right)\left(\sum_{j=1}^{n}𝟙\left\{x_{t}^{\omega }\in A_{j}\right\}\gamma_{j}^{p}f_{j}^{p,c}\left({\underline{x}}_{t}^{\tau }\right)\right)\\ =\;\;\;\; & \sum_{i=1}^{n}\sum_{j=1}^{n}𝟙\left\{x_{t}^{\omega }\in A_{i}\right\}𝟙\left\{x_{t}^{\omega }\in A_{j}\right\}\gamma_{i}^{L}\gamma_{j}^{p}L_{{\underline{\hat{z}}}_{t}}^{c}\left({\underline{x}}_{t}^{\tau }|x_{t}^{\omega }=\beta_{i}\right)f_{j}^{p,c}\left({\underline{x}}_{t}^{\tau }\right)\\ =\;\;\;\; & \sum_{i=1}^{n}𝟙\left\{x_{t}^{\omega }\in A_{i}\right\}\gamma_{i}^{L}\gamma_{i}^{p}L_{{\underline{\hat{z}}}_{t}}^{c}\left({\underline{x}}^{\tau }|x^{\omega }=\beta_{i}\right)f_{i}^{p,c}\left({\underline{x}}^{\tau }\right)\;. \end{array}$$

Then, we use (A3) and (15), leading to

(20) $$\begin{array}{cc} {\overset{˘}{f}}^{e}\left({\underline{x}}_{t}^{\tau },x_{t}^{\omega }\right)\overset{\left(A3\right)}{=} & \sum_{i=1}^{n}𝟙\left\{x_{t}^{\omega }\in A_{i}\right\}\gamma_{i}^{L}\gamma_{i}^{p}f_{N}\left({\underline{x}}_{t}^{\tau };{\underline{\mu }}_{i}^{L},C_{i}^{L}\right)f_{N}\left({\underline{x}}_{t}^{\tau };{\underline{\mu }}_{i}^{p},C_{i}^{p}\right)\\ \overset{\left(15\right)}{=}\; & \sum_{i=1}^{n}𝟙\left\{x_{t}^{\omega }\in A_{i}\right\}\underset{{\overset{˘}{\gamma }}_{i}}{\underbrace{\gamma_{i}^{L}\gamma_{i}^{p}f_{N}\left({\underline{\mu }}_{i}^{p};{\underline{\mu }}_{i}^{L},C_{i}^{L}+C_{i}^{p}\right)}}f_{N}\left({\underline{x}}_{t}^{\tau };{\underline{\mu }}_{i}^{e},C_{i}^{e}\right) \end{array}$$

with

(21) $$\begin{array}{cc} C_{i}^{e} & ={\left({\left(C_{i}^{L}\right)}^{-1}+{\left(C_{i}^{p}\right)}^{-1}\right)}^{-1}\;, \end{array}$$

(22) $$\begin{array}{cc} {\underline{\mu }}_{i}^{e} & =C_{i}^{e}\left({\left(C_{i}^{L}\right)}^{-1}{\underline{\mu }}_{i}^{L}+{\left(C_{i}^{p}\right)}^{-1}{\underline{\mu }}_{i}^{p}\right)\;. \end{array}$$

As the parameters of the unnormalized posterior density, we thus obtain the parameters of the Gaussians according to (21) and (22) and the grid values using

(23) $${\overset{˘}{\gamma }}_{i}^{e}=\gamma_{i}^{L}\gamma_{i}^{p}f_{N}\left({\underline{\mu }}_{i}^{p};{\underline{\mu }}_{i}^{L},C_{i}^{L}+C_{i}^{p}\right)\;.$$

The parameters of the normalized posterior density can be calculated as explained in Section 2.2 using ${\underline{\gamma }}^{e}={\underline{\overset{˘}{\gamma }}}^{e}/\left(2\pi {∥{\underline{\overset{˘}{\gamma }}}^{e}∥}_{1}\right)$. The Gaussians’ parameters are unchanged by the normalization. From the formulae, we can see that the update step has a run time in O(n).

No negative values can result from the formulae for the update step. However, it is possible that all new grid values are zero. In this case, using a higher grid resolution or employing a different filter should be attempted.

### 3.2. Prediction Step

The prediction step is based on a description of the system model in the form of a transition density $f_{t}^{T}\;\left({\underline{x}}_{t+1}^{\tau },x_{t+1}^{\omega }|{\underline{x}}_{t}^{\tau },x_{t}^{\omega }\right)$, which describes the probability of transitioning to state $\left[{\underline{x}}_{t+1}^{\tau };x_{t+1}^{\omega }\right]$ when the current state is $\left[{\underline{x}}_{t}^{\tau };x_{t}^{\omega }\right]$. The foundation for describing the predicted density for the next time step is the Chapman–Kolmogorov equation

(24) $$f_{t+1}^{p}\;\left({\underline{x}}_{t+1}^{\tau },x_{t+1}^{\omega }|{\underline{\hat{z}}}_{1},\ldots ,{\underline{\hat{z}}}_{t}\right)\;=\;\int_{\;R^{2}}\int_{S}\;\underset{f_{t}^{j}\;\left({\underline{x}}_{t+1}^{\tau },x_{t+1}^{\omega },{\underline{x}}_{t}^{\tau },x_{t}^{\omega }\right)}{\underbrace{f_{t}^{T}\;\left({\underline{x}}_{t+1}^{\tau },x_{t+1}^{\omega }|{\underline{x}}_{t}^{\tau },x_{t}^{\omega }\right)f_{t}^{e}\;\left({\underline{x}}_{t}^{\tau },x_{t}^{\omega }|{\underline{\hat{z}}}_{1},\ldots ,{\underline{\hat{z}}}_{t}\right)}}\;dx_{t}^{\omega }d{\underline{x}}_{t}^{\tau }$$

for our state space. The formula on the right-hand side can be implemented as two consecutive operations. The first is to obtain the joint density $f_{t}^{j}$ and the second is to marginalize $x_{t}^{\omega }$ and ${\underline{x}}_{t}^{\tau }$ out.

We now approximate the transition density using a suitable parametric density. As in the update step, we omit the time indices for the functions (refer to (24) for these) and the measurements on which the density is conditioned. We split the transition density into a marginalized density for the periodic part and a conditional density according to

(25) $$f^{T}\;\left({\underline{x}}_{t+1}^{\tau },x_{t+1}^{\omega }|{\underline{x}}_{t}^{\tau },x_{t}^{\omega }\right)=f^{T,c,\mathrm{full}}\;\left({\underline{x}}_{t+1}^{\tau }|x_{t+1}^{\omega },{\underline{x}}_{t}^{\tau },x_{t}^{\omega }\right)f^{T,\omega ,\mathrm{full}}\;\left(x_{t+1}^{\omega }|{\underline{x}}_{t}^{\tau },x_{t}^{\omega }\right)\;.$$

To arrive at a feasible prediction step, we now assume that the orientation at time step t+1 is conditionally independent of the position at time step *t* given the orientation at time step *t*. When this is the case, a transition density $f^{T,\omega }\;\left(x_{t+1}^{\omega }|x_{t}^{\omega }\right)$ may be directly available. If only the full transition density is available, one can obtain $f^{T,\omega }$ by marginalizing the position at time step t+1 out of $f^{T}$ and discarding the (irrelevant) dependency on the position at time step *t*. When the conditional independence does not hold, one may consider conditioning on a specific value for the position part, e.g., the current estimate of the filter or the mean vector in the respective area.

The key idea of the prediction step is to consider the transition density as a function (not a density) of $S\times R^{2}\times S\times R^{2}$. By reordering the parameters, we can see it as a function of $S\times S\times R^{2}\times R^{2}$ Then, we can consider the toroidal manifold S×S as the periodic domain that we subdivide. For compatibility with $f^{e}$, we subdivide S×S equally along both axes so that we obtain the areas ${\left\{A_{i}\times A_{j}\right\}}_{\left(i,j\right)\in {\left\{1,\ldots ,n\right\}}^{2}}$. For each area $A_{i}\times A_{j}$, the corresponding grid point is $\left[\beta_{i};\beta_{j}\right]$. Then, we can get a similar grid-based representation as previously discussed by introducing some assumptions that resemble (A1)–(A3). Combined with the assumption introduced in the previous paragraph, we arrive at the list of assumptions

(B1) $f^{T,\omega }\;\left(x_{t+1}^{\omega }|x_{t}^{\omega }\right)=f^{T,\omega ,\mathrm{full}}\;\left(x_{t+1}^{\omega }|{\underline{x}}_{t}^{\tau },x_{t}^{\omega }\right)$ holds for all ${\underline{x}}_{t}^{\tau }\in R^{2}$.
(B2) The function value of $f^{T,\omega }\;\left(x_{t+1}^{\omega }|x_{t}^{\omega }\right)$ in each area in S×S is identical to the grid value for this area, i.e., $f^{T,\omega }\;\left(\alpha_{1}|\alpha_{2}\right)=f^{T,\omega }\;\left(\beta_{i}|\beta_{j}\right)=\gamma_{i,j}^{T}$ for all $\alpha_{1}\in A_{i}$, $\alpha_{2}\in A_{j}$.
(B3) The conditional density $f^{T,c,\mathrm{full}}\left({\underline{x}}_{t+1}^{\tau }|\alpha_{1},{\underline{x}}_{t}^{\tau },\alpha_{2}\right)$ is the same function for all $\alpha_{1}\in A_{i}$, $\alpha_{2}\in A_{j}$ (we denote it by $f_{i,j}^{T,c}\left({\underline{x}}_{t+1}^{\tau }|{\underline{x}}_{t}^{\tau }\right)$).
(B4) Each conditional density can be written as
  (26) $$f_{i,j}^{T,c}\left({\underline{x}}_{t+1}^{\tau }|{\underline{x}}_{t}^{\tau }\right)=f_{N}\left({\underline{x}}_{t+1}^{\tau };F_{t,i,j}{\underline{x}}_{t}^{\tau }+{\underline{\hat{u}}}_{t,i,j},C_{t,i,j}^{\underline{w}}\right)$$
  and we have all relevant system matrices $F_{t,i,j}$, inputs ${\underline{\hat{u}}}_{t,i,j}$, and covariance matrices $C_{t,i,j}^{\underline{w}}$.

With (B3), we assume that for a fixed orientation, the transitional part follows a linear system model. Writing it using random vectors (written in bold font), the model can be described by

(27) $${\underline{x}}_{t+1,i,j}^{\tau }=F_{t,i,j}{\underline{x}}_{t,i,j}^{\tau }+{\underline{\hat{u}}}_{t,i,j}^{\tau }+{\underline{w}}_{t,i,j}^{\tau }$$

with fixed and known system input ${\underline{\hat{u}}}_{t,i,j}^{\tau }$ and system noise ${\underline{w}}_{t,i,j}∼N\left(\underline{0},C_{t,i,j}^{\underline{w}}\right)$. If ${\underline{w}}_{t,i,j}$ is not a Gaussian, we propose to approximate it with one via moment matching. The input ${\underline{\hat{u}}}_{t,i,j}^{\tau }$ can be different for each area and can thus depend on the orientation. If it is a function in the form of $g(x_{t}^{\omega })$, one can use $\beta_{i}$ as $x_{t}^{\omega }$, i.e., ${\underline{\hat{u}}}_{t,i,j}^{\tau }=g\left(\beta_{j}\right)$. Similarly, the input can also be a function of $x_{t}^{\omega }$ and $x_{t+1}^{\omega }$. While we never explicitly consider inputs for the orientation part, they can be considered by the nonlinear system model described by $f^{T,\omega }$.

In our description of the transition density, we shall from now on omit the time indices of the input vector and the system and covariance matrices, which is always *t*. Based on its parameters, the transition density is given by

(28) $$\begin{array}{cc} & f^{T}\;\left({\underline{x}}_{t+1}^{\tau },x_{t+1}^{\omega }|{\underline{x}}_{t}^{\tau },x_{t}^{\omega };{\left\{\gamma_{i,j}^{T},F_{i,j},{\underline{\hat{u}}}_{i,j}^{\tau },C_{i,j}^{\underline{w}}\right\}}_{\left(i,j\right)\in {\left\{1,\ldots ,n\right\}}^{2}}\right)\\ \overset{\left(B1)-(B2\right)}{=} & \sum_{i=1}^{n}\sum_{j=1}^{n}𝟙\left\{x_{t+1}^{\omega }\in A_{i}\right\}𝟙\left\{x_{t}^{\omega }\in A_{j}\right\}\gamma_{i,j}^{T}f^{T,c,\mathrm{full}}\left({\underline{x}}_{t+1}^{\tau }|x_{t+1}^{\omega },{\underline{x}}^{\tau },x_{t}^{\omega }\right)\\ \overset{\left(B3\right)}{=}\;\;\; & \sum_{i=1}^{n}\sum_{j=1}^{n}𝟙\left\{x_{t+1}^{\omega }\in A_{i}\right\}𝟙\left\{x_{t}^{\omega }\in A_{j}\right\}\gamma_{i,j}^{T}f_{i,j}^{T,c}\left({\underline{x}}_{t+1}^{\tau }|{\underline{x}}_{t}^{\tau }\right)\;. \end{array}$$

Due to potential approximations involved, the density may be unnormalized. By default, we renormalize after the prediction step (if necessary) and do not perform any normalization on the parametric transition density.

We start our derivation of the predicted density by writing out and reformulating the joint density as

(29) $$\begin{array}{cc} & \begin{array}{cc} & f^{j}\;\left({\underline{x}}_{t+1}^{\tau },x_{t+1}^{\omega },{\underline{x}}_{t}^{\tau },x_{t}^{\omega }\right)\\ \overset{\left(\mathrm{B2})\&(\mathrm{B3}\right)}{=} & \left(\sum_{i=1}^{n}\sum_{j=1}^{n}𝟙\left\{x_{t+1}^{\omega }\in A_{i}\right\}𝟙\left\{x_{t}^{\omega }\in A_{j}\right\}\gamma_{i,j}^{T}f_{i,j}^{T,c}\left({\underline{x}}_{t+1}^{\tau }|{\underline{x}}_{t}^{\tau }\right)\right)\left(\sum_{k=1}^{n}𝟙\left\{x_{t}^{\omega }\in A_{k}\right\}\gamma_{k}^{e}f_{k}^{e}\left({\underline{x}}_{t}^{\tau }\right)\right) \end{array}\\ & \;\begin{array}{cc} = & \sum_{i=1}^{n}\sum_{j=1}^{n}\sum_{k=1}^{n}𝟙\left\{x_{t+1}^{\omega }\in A_{i}\right\}𝟙\left\{x_{t}^{\omega }\in A_{j}\right\}𝟙\left\{x_{t}^{\omega }\in A_{k}\right\}\gamma_{i,j}^{T}\gamma_{k}^{e}f_{i,j}^{T,c}\left({\underline{x}}_{t+1}^{\tau }|{\underline{x}}_{t}^{\tau }\right)f_{k}^{e}\left({\underline{x}}_{t}^{\tau }\right)\\ = & \sum_{i=1}^{n}\sum_{j=1}^{n}𝟙\left\{x_{t+1}^{\omega }\in A_{i}\right\}𝟙\left\{x_{t}^{\omega }\in A_{j}\right\}\gamma_{i,j}^{T}\gamma_{j}^{e}f_{i,j}^{T,c}\left({\underline{x}}_{t+1}^{\tau }|{\underline{x}}_{t}^{\tau }\right)f_{j}^{e}\left({\underline{x}}^{\tau }\right)\;. \end{array} \end{array}$$

We define $\gamma_{i,j}^{j}=\gamma_{i,j}^{T}\gamma_{j}^{e}$ and marginalize all components of the state at time step *t* out to obtain the predicted density

(30) $$\begin{array}{cc} & f_{t+1}^{p}\;\left({\underline{x}}_{t+1}^{\tau },x_{t+1}^{\omega }\right)\\ = & \int_{S}\int_{R^{2}}\sum_{i=1}^{n}\sum_{j=1}^{n}𝟙\left\{x_{t+1}^{\omega }\in A_{i}\right\}𝟙\left\{x_{t}^{\omega }\in A_{j}\right\}\gamma_{i,j}^{j}f_{i,j}^{T,c}\left({\underline{x}}_{t+1}^{\tau }|{\underline{x}}_{t}^{\tau }\right)f_{j}^{e}\left({\underline{x}}_{t}^{\tau }\right)d{\underline{x}}_{t}^{\tau }dx_{t}^{\omega }\\ = & \sum_{i=1}^{n}𝟙\left\{x_{t+1}^{\omega }\in A_{i}\right\}\sum_{j=1}^{n}\int_{R^{2}}\gamma_{i,j}^{j}f_{i,j}^{T,c}\left({\underline{x}}_{t+1}^{\tau }|{\underline{x}}_{t}^{\tau }\right)f_{j}^{e}\left({\underline{x}}_{t}^{\tau }\right)d{\underline{x}}^{\tau }\int_{S}𝟙\left\{x_{t}^{\omega }\in A_{j}\right\}dx_{t}^{\omega }\\ = & \sum_{i=1}^{n}𝟙\left\{x_{t+1}^{\omega }\in A_{i}\right\}\left|A_{i}\right|\sum_{j=1}^{n}\int_{R^{2}}\gamma_{i,j}^{j}f_{i,j}^{T,c}\left({\underline{x}}_{t+1}^{\tau }|{\underline{x}}_{t}^{\tau }\right)f_{j}^{e}\left({\underline{x}}_{t}^{\tau }\right)dx_{t}^{\omega }\;. \end{array}$$

We now introduce the assumption (B4) and use that the value of the pdf of a Gaussian at a point only depends on the distance between the point and the mode to rearrange the parameters of the first Gaussian. This allows us to employ (15). Thus, we get

(31) ft+1px_t+1τ,xt+1ω=(B4)∑i=1n𝟙xt+1ω∈AiAi∑j=1n∫R2γi,jjfNx_t+1τ;Fi,jx_tτ+u^_i,jτ,Ci,jw_fNx_tτ;μ_je,Cjedx_tτ=∑i=1n𝟙xt+1ω∈AiAi∑j=1n∫R2γi,jjfNFi,jx_tτ;x_t+1τ−u^_i,jτ,Ci,jw_fNx_tτ;μ_je,Cjedx_tτ=(15)∑i=1n𝟙xt+1ω∈AiAi∑j=1n∫R2γi,jjfNx_t+1τ−u^_i,jτ;Fi,jμ_je,Ci,jw_+Fi,jCjeFi,j⊤∑i=1n𝟙xt+1ω∈AiAi∑j=1n∫R2γi,jjfNx_tτ;μ_q,Cqdx_tτ,

in which the precise values of ${\underline{\mu }}^{q}$ and $C^{q}$ are irrelevant. Since $f_{N}\left({\underline{x}}_{t}^{\tau };{\underline{\mu }}^{q},C^{q}\right)$ is the only term that depends on ${\underline{x}}_{t}^{\tau }$, we can push back the integral sign to that term. Then, it is evident that the integral is 1 because we integrate a Gaussian density over the whole domain.

By adapting the parameters of the remaining Gaussian density in a way that does not change the function value, we obtain

(32) $$f_{t+1}^{p}\;\left({\underline{x}}_{t+1}^{\tau },x_{t+1}^{\omega }\right)=\sum_{i=1}^{n}𝟙\left\{x_{t+1}^{\omega }\in A_{i}\right\}\left|A_{i}\right|\sum_{j=1}^{n}\gamma_{i,j}^{j}f_{N}\left({\underline{x}}_{t+1}^{\tau };F_{i,j}{\underline{\mu }}_{j}^{e},+{\underline{\hat{u}}}_{i,j}^{\tau },C_{i,j}^{\underline{w}}+F_{i,j}C_{j}^{e}F_{i,j}^{⊤}\right)\;.$$

Finally, we pull the factor ${∥{\underline{\gamma }}_{i,:}^{j}∥}_{1}=\sum_{k=1}^{n}\gamma_{i,k}^{j}$ out of the second sum to obtain

(33) $$f_{t+1}^{p}\;\left({\underline{x}}_{t+1}^{\tau },x_{t+1}^{\omega }\right)=\sum_{i=1}^{n}𝟙\left\{x_{t+1}^{\omega }\in A_{i}\right\}\overset{\gamma_{i}^{p}}{\overbrace{\left|A_{i}\right|{∥{\underline{\gamma }}_{i,:}^{j}∥}_{1}}}\;\sum_{j=1}^{n}\frac{\gamma_{i,j}^{j}}{{∥{\underline{\gamma }}_{i,:}^{j}∥}_{1}}f_{N}\left({\underline{x}}_{t+1}^{\tau };F_{i,j}{\underline{\mu }}_{j}^{e}+{\underline{\hat{u}}}_{i,j}^{\tau },C_{i,j}^{\underline{w}}+F_{i,j}C_{j}^{e}F_{i,j}^{⊤}\right).$$

This is already reminiscent of the desired form (3). The factors in front of the second sum can be directly combined into the grid values for the predicted density ${\underline{\gamma }}^{p}$. However, instead of a single Gaussian, we now have a mixture of Gaussians on $R^{2}$ with the weights $\gamma_{i,j}^{j}/{∥{\underline{\gamma }}_{i,:}^{j}∥}_{1}$.

To prevent an increase in the number of parameters and an increase in the complexity of the filter over time, we perform mixture reduction. In our current implementation, we always reduce the mixture to a single Gaussian using moment matching. However, it would also be possible to perform a different reduction and preserve more components with alternative approaches [27]. Our use of moment matching is motivated by its speed and ease of implementation. Moment matching yields

(34) $${\underline{\mu }}_{i}^{p}=\sum_{j=1}^{n}\frac{\gamma_{i,j}^{j}}{{∥{\underline{\gamma }}_{i,:}^{j}∥}_{1}}\left(F_{i,j}{\underline{\mu }}_{j}^{e}+{\underline{\hat{u}}}_{i,j}^{\tau }\right)$$

for the predicted mean of the *i*th area. For the covariance, we need to include both the part stemming from the individual covariances and the additional part caused by the differences in the means. For the area *i*, we obtain the formula

(35) $$C_{i}^{p}=\left(\sum_{j=1}^{n}\frac{\gamma_{i,j}^{j}}{{∥{\underline{\gamma }}_{i,:}^{j}∥}_{1}}\left(C_{i,j}^{\underline{w}}+F_{i,j}C_{j}^{e}F_{i,j}^{⊤}\right)\right)+\mathrm{SampleCov}\left({\left\{F_{i,j}{\underline{\mu }}_{j}^{e}+{\underline{\hat{u}}}_{i,j}^{\tau }-{\underline{\mu }}_{i}^{p}\right\}}_{j=1}^{n},\frac{\gamma_{i,j}^{j}}{{∥{\underline{\gamma }}_{i,:}^{j}∥}_{1}}\right)\;,$$

with SampleCov denoting the sample covariance of the weighted zero mean (note that we subtract ${\underline{\mu }}_{i}^{p}$) sample set. For a set of vectors $\left\{{\underline{v}}_{1},\ldots ,{\underline{v}}_{n}\right\}$ and a vector of weights $\left[w_{1},\ldots ,w_{n}\right]$, we obtain the values of the sample covariance according to

$$c_{j,k}^{\mathrm{SampleCov}}=\sum_{l=1}^{n}w_{l}v_{j,l}v_{k,l}\;,$$

in which $v_{j,l}$ is the *l*th component of the vector ${\underline{v}}_{j}$. Determining this sum is in O(n). It has to be determined for all 3 (unique) entries of the covariance matrix.

Since both the mean and the covariance can be determined in O(n), the total effort of the mixture reduction is in O(n). Since *n* mixture reductions are required, the total effort involved to determine all new means and covariances is in $O(n^{2})$. Calculating each of the *n* entries of ${\underline{\gamma }}^{p}$ also involves summing over *n* values, and thus, the calculation of the grid values is also in $O(n^{2})$. With both operations being in $O(n^{2})$, the total effort of the prediction step is in $O(n^{2})$.

## 4. Evaluation

In our evaluation, we consider a tracking scenario on SE(2) and compare our novel S3F with a particle filter (PF), the progressive SE(2) Bingham filter (ProgSE2BF) [14], and the unscented Kalman filter for manifolds (UKF-M).

The PF, ProgSE2BF, and S3F are part of the latest version of libDirectional [28]. For the UKF-M, we use the implementation of [13]. The UKF-M involves a parameter that can be tuned, which is called α in [29]. This parameter influences how far away from the mean the sigma points of the UKF [30] are placed. [13] recommends values in the range [0.001, 1]. The implementation allows specifying different parameters for the state uncertainty, the uncertainty introduced in the prediction step, and the measurement uncertainty. We set all entries of the vector to the same value and considered the values 0.001, 0.01, 0.1, and 1. While the results were similar for the different parameters, using 1 led to the best results. Therefore, we used this parameter in our evaluation.

### 4.1. Scenario Description

In the considered scenario, we track a vehicle for 50 time steps. Its initial position is distributed according to ${\underline{x}}_{1}^{\tau }∼N\left(\underline{0},I\right)$ (with $\underline{0}$ denoting a zero vector and I an identity matrix), and its orientation (describing its heading angle) $x_{1}^{\omega }$ is distributed according to a von Mises distribution (Section 3.5.4, [25]) with μ=0 and κ=1 (i.e., $x_{1}^{\omega }∼\mathrm{VM}\left(0,1\right)$) in each run.

**Figure 5:**
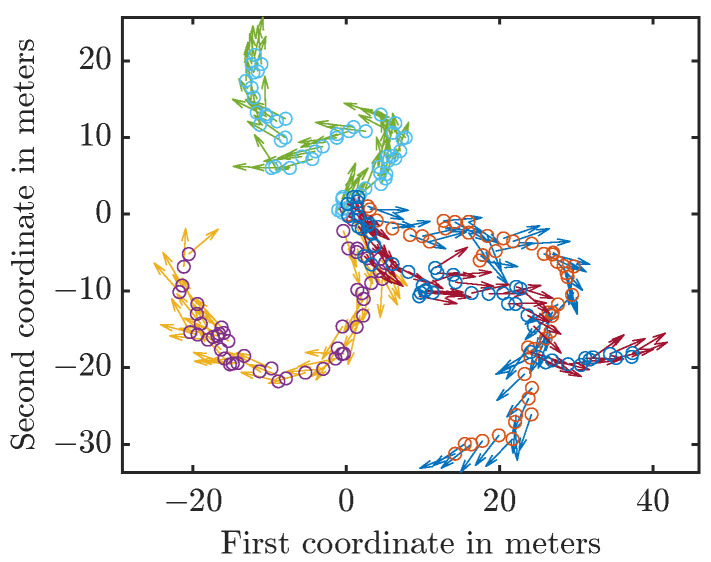
Sample trajectories. The circles indicate the positions, the arrows the heading angles.

The vehicle moves approximately one unit per time step in the direction it is facing. Random noise is applied to both the position and the orientation. The system model is thus

(36) $$\left[\begin{array}{c} x_{t+1}^{\omega }\\ {\underline{x}}_{t+1}^{\tau } \end{array}\right]=\left[\begin{array}{c} x_{t}^{\omega }+{\underline{w}}_{t}^{\omega }\;\mathrm{mod}\;2\pi \\ {\underline{x}}_{t}^{\tau }+\left[\begin{array}{c} \cos(x_{t}^{\omega })\\ \sin(x_{t}^{\omega }) \end{array}\right]+{\underline{w}}_{t}^{\tau } \end{array}\right]$$

with ${\underline{w}}_{t}^{\tau }∼N\left(\underline{0},I\right)$ and $w_{t}^{\omega }∼\mathrm{VM}\left(0,10\right)$ for all t∈N. The uncertainty in the orientation influences the uncertainty in the position as $x_{t}^{\omega }$ influences ${\underline{x}}_{t+1}^{\tau }$. Thus, while ${\underline{w}}_{t}^{\tau }$ and $w_{t}^{\omega }$ are independent, the noise in the angle influences later positions as ${\underline{w}}_{t}^{\omega }$ influences $x_{t+1}^{\omega }$, which in turn influences ${\underline{x}}_{t+2}^{\tau }$. In each run, we drew the initial state from the initial prior and then generated the new states by applying the system model and sampling from the involved distributions. Thus, the path was different for each run. We provide four sample trajectories in Figure 5.

We can only measure the position and not the heading angle of the vehicle. The measurement model is ${\underline{z}}_{t}={\underline{x}}_{t}^{\tau }+{\underline{v}}_{t}$ with ${\underline{v}}_{t}∼N\left(\underline{0},0.5\;\cdot \;I\right)$ for all t∈N. This makes it challenging for filters since they have to derive information about the heading angle based on the dependency of the heading angle and position over time.

### 4.2. Models Used by the Filters

In this subsection, we describe how we applied the filters to the considered scenario. If possible, required conversions are done offline once and the results are used for all runs of the filter.

For the initialization of the PF with *n* particles, ${\underline{x}}_{1}^{\tau }$ and $x_{1}^{\omega }$ were sampled *n* times. These samples were then used as equally weighted samples for the state representation of the PF. For the ProgSE2BF, we draw 100,000 samples from ${\underline{x}}_{1}^{\tau }$ and $x_{1}^{\omega }$ and fit a density for dual quaternions states [31] to the samples.

Due to the independence of the distributions for the initial position and angle, we can directly store the function values on the grid in the vector ${\underline{\gamma }}_{1}^{p}$ and initialize all Gaussians with ${\underline{\mu }}_{1,i}^{p}=\underline{0}$ and $C_{1,i}^{p}=I$ for i∈{1,…,n} for the S3F. For the UKF-M, we approximate the von Mises distribution with a wrapped normal (WN) distribution (Section 3.5.7, [25]) $\mathrm{WN}(0,\sigma_{1}^{p,\omega })$ via moment matching. The covariance matrix for the UKF-M is then a block diagonal matrix with the squared standard deviation ${\left(\sigma_{1}^{p,\omega }\right)}^{2}$ of the WN and the covariance matrix I for the position on the diagonal.

Concerning the system model, we can sample the noise distributions for the PF and apply (36) (with samples instead of random variables) to the samples to obtain the samples of the predicted density. For the ProgSE2BF, we draw 100,000 samples from the system noise $\left[{\underline{w}}_{t}^{\tau };{\underline{w}}_{t}^{\omega }\right]$ and fit a density for dual quaternion states to it. This has to be done only once offline because the resulting density can be used in all runs and time steps.

For the S3F, we evaluate $\mathrm{VM}(x_{t+1}^{\omega };x_{t}^{\omega },1)$ on the two-dimensional grid to obtain the grid values $\gamma_{i,j}^{T}=\mathrm{VM}\left(\beta_{i};\beta_{j},1\right)$. This is done in a precomputation step offline. As the input ${\underline{\hat{u}}}_{t,i,j}^{\tau }$, we use $\left[\cos\left(\beta_{j}\right);\sin\left(\beta_{j}\right)\right]$, which describes the change in the position in one time step for the orientation $\beta_{j}$.

For the UKF-M, we use an existing model for tracking on SE(2) for scenarios involving gyroscopes. We provide a gyroscope reading of 1 m per time step (which is the true velocity when disregarding noise) to the model. For the system noise, we match $w_{t}^{\omega }$ with a WN distribution $\mathrm{WN}(0,\sigma^{w,\omega })$ and provide $\sigma^{w,\omega }$ to the filter. In the gyroscope-based model, the position noise needs to be given as longitudinal and transversal shifts. While our uncertainty in the position is given relative to the world coordinate system, our system covariance matrix for the position is I, and this covariance matrix does not change under rotations of the coordinate system. Thus, we use I as the system covariance for the position for the UKF-M.

All filters except the UKF-M use likelihood functions. In the PF, the particle weights are multiplied with the function values of the likelihood at the particle positions. Afterward, a resampling step is performed. For the ProgSE2BF, we use the progressive update step that uses a likelihood function. The parameter for the progression (called τ in [14]) is set to 0.02. For the S3F, all grid values $\gamma_{i}$ are set to 1 since the measurement does not provide any information on the orientation. The measurement ${\underline{\hat{z}}}_{t}$ is used as the mean ${\underline{\mu }}_{i}^{L}$ for all Gaussians and 0.5·I is used for all covariances $C_{i}^{L}$. A measurement function that extracts the state from the position is provided to the UKF-M. The employed covariance matrix is the covariance matrix of the measurement noise, i.e., 0.5·I.

### 4.3. Evaluation Metrics

For all filters, we measured the run times and divided them by the number of time steps to provide the run time per time step. The run times were measured on an Intel Xeon Gold 6230 running at 2.1 GHz with 3 GB of RAM allocated to the job. Matlab 2021a ran with a single thread on Red Hat Enterprise Linux 8.2.

To assess the estimation quality of the filters, we consider the accuracy of the position and orientation estimates. We consider them separately since one would have to specify a weighting factor when combining them, and any chosen value may appear arbitrary. Multiple runs were performed, and the errors and run times of all individual runs were condensed into a single value by determining their average.

For the PF, we discarded the part of the vector describing the orientation to marginalize it out and then determined the mean of the particles to obtain the position estimate. We did this likewise for the orientation component using the mean direction (Section 2.2.1, [25]) instead of the mean. For the S3F, we determined the mean of the linear part by marginalizing out the orientation part. This can be done analytically by determining the vector $\underline{\rho }={\underline{\gamma }}_{50}^{e}/{∥{\underline{\gamma }}_{50}^{e}∥}_{1}$, weighting the Gaussians according to $\underline{\rho }$, and finally extracting the mean of the Gaussian mixture (see (34)). The circular mean of the weighted grid points is used as the estimate for the orientation.

The average Euclidean distance between the true and the estimated position is used as the position error. As error criterion for the periodic part, we use the topology-aware distance (1). For both the position and orientation, we compare the estimation accuracy after the update step. Since the position (and thus the measurement) is influenced by the previous orientation, it contains information on it. However, no information on the change from the previous to the current time step can be obtained. Hence, the accuracy of the orientation estimate is inherently limited by the uncertainty in the system evolution from the previous to the current time step. Therefore, not even a noise-free position measurement could lead to an elimination of all uncertainty in the orientation component.

We will also consider how often the filters failed during the evaluation. The PF can fail when all particle weights are zero after an update step. Since the likelihood is nonzero everywhere, this can only happen due to numerical imprecision (double precision was used). The S3F could potentially fail if all grid points are 0 in some time step.

Based on these criteria, we performed two evaluations. In the first, we extracted the estimates in all time steps and considered the error over the course of the time steps. While the filter results were highly variable in a single run, we also determined the averages over multiple runs to see whether the filter performance degrades over time or stays constant. This evaluation also showed us when the effect of the prior density had mostly vanished for the tracker. We chose to use only 100 particles for the PF and 15 areas for the S3F for this evaluation to see if the filters tend to diverge for low numbers of particles or areas.

In the second evaluation, we aimed to assess the accuracy of the filters during longer tracking tasks. For this, we chose to only consider the accuracy at the 50th time step. Our first evaluation showed that the filters had reached a stable accuracy by then. We did not average over the errors in all times steps to eliminate the effect of the first time steps, in which the prior density still influences the filter result or the filter has not yet reached a stable accuracy. Further, averaging over the errors in all time steps would have made us average over correlated errors. The run times are only considered in the second evaluation. We did not extract an estimate in every time step in this evaluation, and thus, the run times measured exclude the extraction of an estimate.

### 4.4. Evaluation Results

In the first subsection, we provide the results of our first evaluation, in which we only considered specific configurations for all filters. The results of the second evaluation, in which we considered different numbers of particles for the PF and areas for the S3F, are given in the second subsection.

#### 4.4.1. Evaluation Considering All Time Steps

**Figure 6:**
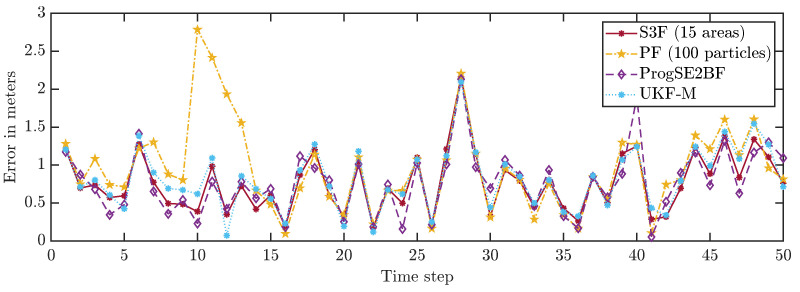
Position errors in all time steps in an example run.

**Figure 7:**
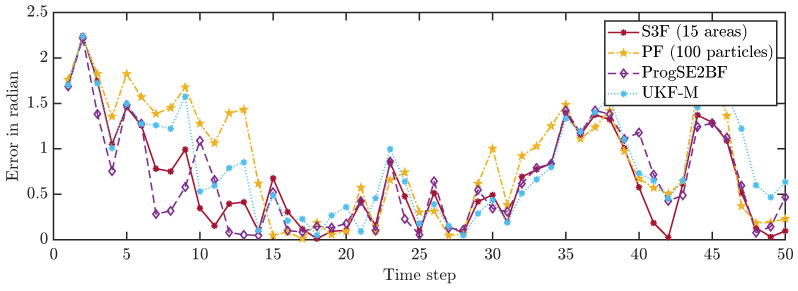
Orientation errors in all time steps in an example run.

We first consider a single run and depict the position errors in Figure 6 and the orientation errors in Figure 7. We can see that the error goes up and down over time, which is what we had expected. While most filters work well on average, the system can evolve in a much different way than we expect it to do on average due to the effects of the random variables in the system model. Furthermore, with the considered Gaussian measurement noise, the obtained measurements in each time step can have arbitrarily large errors, just with small errors being more likely than large errors. From the results for a single run, it is not evident which filter performs best. It is merely evident that the PF with 100 particles once goes off track in its position estimate and appears to be worse overall.

**Figure 8:**
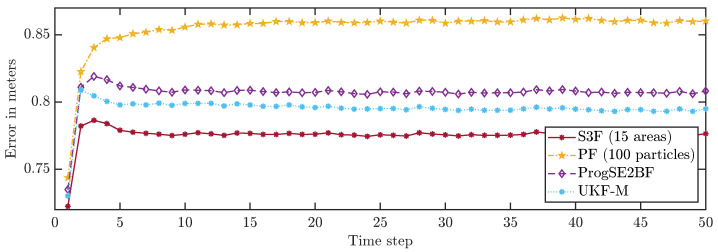
Average position errors in all time steps.

**Figure 9:**
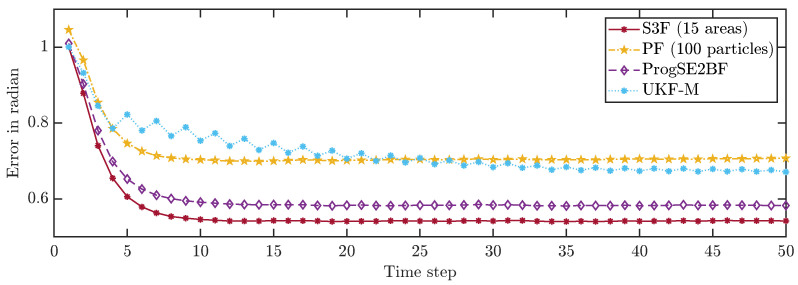
Average orientation errors in all time steps.

To allow for a better comparison of the filters, we averaged the errors in all time steps over 200,000 runs. The results are shown in Figure 8 and Figure 9. None of the considered filters failed in any of the runs. We can see that the S3F with 15 areas performed best both concerning the position and the orientation estimates. The UKF-M yielded better position estimates than the ProgSE2BF. However, the ProgSE2BF provided better orientation estimates. The PF only outperformed the UKF-M in the beginning and was the worst filter at the end of the run. We can see that the UKF-M is slow to converge to a stable quality. When it does, it performs better than the PF with 100 particles. However, as we will see in the next section, the PF can provide better estimates than both the UKF-M and the ProgSE2BF given a sufficient number of samples. Due to the high number of runs, the oscillating behavior of the UKF-M in the error in the orientation component is certain not to be purely noise and may be attributed to its suboptimal handling of the information on the orientation. An important observation for the second evaluation is that it is fair to compare the filters at the 50th time step if one wants to analyze the long-term accuracy of the filters.

#### 4.4.2. Evaluation of the Last Estimates and Run Times

The filters were evaluated based on 70,000 runs in this evaluation. Several configurations were considered for the PF and S3F. The PF with 5 particles failed in 7014 runs and the PF with 11 particles in 145 runs. No failures were observed for all the other configurations and filters.

**Figure 10:**
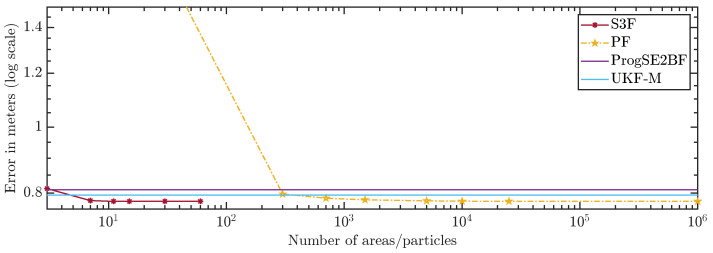
Position error over number of areas or particles.

**Figure 11:**
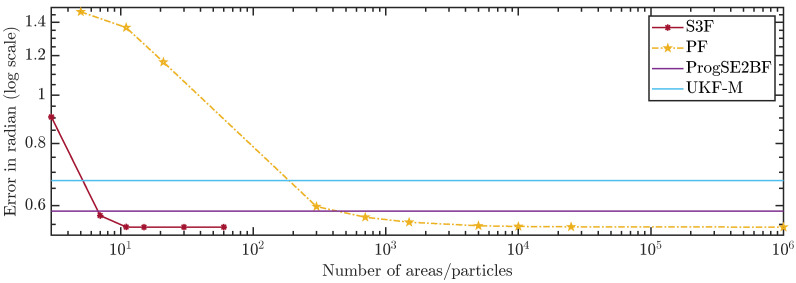
Orientation error over number of areas or particles.

In Figure 10 and Figure 11, we show the average errors over the number of areas or particles. The ProgSE2BF and UKF-M have a fixed number of parameters and are therefore shown as straight lines. Using only 15 areas, the S3F achieved a higher mean accuracy than a PF with 1,000,000 particles. Using 7 areas, the S3F achieved better accuracy than the ProgSE2BF for the position and orientation. The UKF-M is better than the S3F with 3 areas but worse than the S3F with 7 areas. The PF achieves a better accuracy than the ProgSE2BF when using 700 particles. Using 15 areas, the S3F already achieved very high accuracy. The difference to the accuracy obtained using 30 areas is only in the order of $10^{-6}$. The accuracy of the S3F with 15 areas is also higher (although only slightly) than that of a particle filter using 1,000,000 particles. We believe this accuracy to be close to the accuracy an optimal Bayes filter would achieve in this scenario.

**Figure 12:**
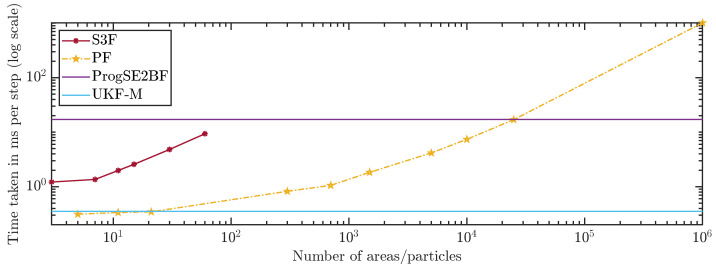
Run time over number of areas or particles.

The run times of the filters are shown in Figure 12. We can see that the S3F is much slower per area than the PF per particle, which was expected. We also observe an expected faster increase in the run time for the S3F. While the PF is in O(n), the S3F is in $O(n^{2})$. The S3F is faster than the ProgSE2BF in all considered configurations. The PF with 25,000 particles had a run time similar to that of the ProgSE2BF but required much more run time with 1,000,000 particles. The UKF-M was faster than all other filters except the PF with 21 or fewer particles.

**Figure 13:**
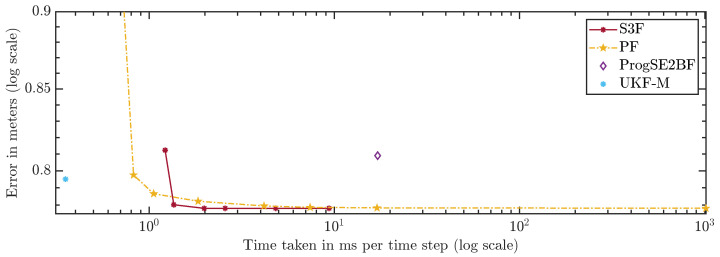
Position error over run time.

**Figure 14:**
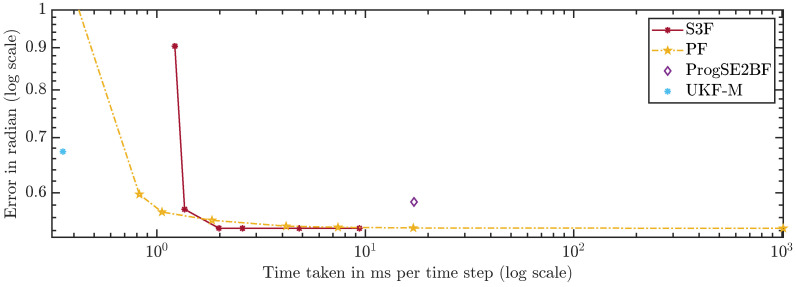
Orientation error over run time.

For a fair comparison of the filters, which have very different run times, we compare the errors over the run times in Figure 13 and Figure 14. A configuration to the lower left of another is strictly better because more accurate results were achieved using less run time. Configurations to the upper left were faster but provided less accurate results. Ones to the lower right were slower but better in terms of accuracy. The latter two cases do not allow for a clear ranking. The plots can be used to assess which filters should be considered given certain run time constraints. However, they can only serve as a rough guideline since the run times highly depend on the scenario, implementation, and hardware.

The S3F using 3 areas is worse than the PF with 300 and 700 particles. However, for configurations of the PF with 5000 particles and more, one can find at least one configuration of the S3F that performs better. In the configuration using 15 areas, the S3F still uses less than 3 ms per time step and is faster and more accurate than the PF with 1,000,000 particles.

Using 7 areas, the S3F achieves better accuracy than the ProgSE2BF at lower run times. The ProgSE2BF is slower than the UKF-M and provides worse position estimates, but its orientation estimates are better, and thus, none of the two filters is clearly superior to the other. The UKF-M is better than the S3F using 3 areas. However, using 7 areas, the S3F yields more accurate results than the UKF-M while taking only less than 1.4 ms per time step. The PF provides more accurate results than the UKF-M in the configuration with 700 particles, in which it takes less than 1.1 ms per time step.

## 5. Discussion, Summary, and Outlook

In the first subsection, we compare our novel filter with other state-of-the-art approaches based on their theoretical properties and evaluation results. The conclusion of our paper is provided in the second subsection. The third subsection concludes the paper with an outlook.

### 5.1. Discussion

While some existing approaches, such as the UKF-M and the ProgSE2BF, use a fixed number of parameters, the number of parameters can be increased to arbitrary numbers in the S3F and the PF. Generally, a higher number of particles in the PF or areas in the S3F leads to better results.

If good results at rapid run times are desired or only very low computation power is available, the UKF-M may be a good choice. The S3F should be preferred over the ProgSE2BF as the S3F can achieve better results at lower run times. Generally, both the PF and S3F can achieve higher accuracy than the UKF-M and the ProgSE2BF. Thus, if sufficient computation power is available, one should rather employ the S3F or PF instead of the UKF-M or ProgSE2BF.

While the run time of the PF only increases linearly in the number of particles, the accuracy of its estimates may converge only slowly. This is also reflected in the evaluation results in Section 4.4. The increase in the run time for the S3F is linear in the update step and quadratic in the prediction step, leading to a total complexity of $O(n^{2})$. However, the convergence of the accuracy is so much faster than for the PF, and almost optimal results were achieved with relatively low run times. Hence, we do not recommend using a PF with a high number of particles but rather an S3F. Another advantage of the S3F is that it is deterministic, while the PF is non-deterministic (unless one uses a fixed seed for the random number generator).

In our evaluation, good results were obtained, even though none of the considered densities fulfilled the assumptions (A1)–(A3) and (B2)–(B4). However, higher numbers of areas may be required for high-quality estimates when the densities are more concentrated.

Different ways to provide a continuous density exist for the S3F. No densities are inherently provided by the PF. A limitation of the S3F is that one has to provide a transition density for the orientation that is conditionally independent of the previous state. If no such transition density can be provided and no good approximation can be generated using information from the filter, one may consider using the PF instead.

### 5.2. Summary

In this paper, we presented a novel filter for SE(2) for which we rewrote the joint density for the full SE(2) state as a marginalized density for the orientation and a conditional density for the position. By subdividing the domain along the periodic dimension into multiple areas, we were able to present an amalgamation of a grid filter and a Kalman filter. While the S3F for SE(2) states requires more operations than a 1-D grid filter for S that permits the use of arbitrary transition densities [20], it is also in $O(n^{2})$ and thus in the same complexity class.

Our filter does not support all kinds of system models in the prediction step. Further, since densities do generally not fulfill the assumptions introduced in this paper, the filter involves approximations. However, in our evaluation, the S3F showed a much better convergence than the PF and achieved a very high accuracy using a low number of areas. Using 15 areas, higher accuracy was achieved than with a PF with 1,000,000 particles. The S3F also outperformed the ProgSE2BF by achieving a slightly higher accuracy using considerably less run time. While not as fast as the UKF-M, the S3F can achieve far higher accuracy.

### 5.3. Outlook

Future work will involve applying the S3F to SE(3) [32] and other manifolds that can be written as a Cartesian product of periodic (or bounded) and linear manifolds. As another possible extension, using ideas of the extended or unscented Kalman filter in the prediction step may help to allow for a certain degree of nonlinearity in the system model for the position.

Throughout this paper, we assumed the system and measurement models to be precisely known. Future work may concern approaches for imprecisely known models. One way to deal with imprecision in the parameters of linear models on linear domains is to employ robust filters [33,34]. For SE(2), even if, e.g., only the measurement model for the position is imprecise, this will have to be also considered for the orientation as the position and orientation are generally dependent.

Finally, adaptive subdivisions may help to keep the number of areas low without sacrificing too much accuracy. The basis for this could be adaptive grids as used in [35]. Reducing the required number of areas is particularly important for higher dimensions, e.g., for estimating the pose of a drone along with sensor drifts, biases, and other relevant quantities.

## Data Availability

The data for all evaluation runs were generated artificially. The code to generate data and links to the data used for this paper are available at https://github.com/KIT-ISAS/Sensors21_S3FforSE2 (accessed on 19 August 2021).
